# Improvement of the CO_2_ Sensitivity: HPTS-Based Sensors along with Zn@SnO_2_ and Sn@ZnO Additives

**DOI:** 10.1021/acsomega.3c03708

**Published:** 2023-07-31

**Authors:** Merve Zeyrek Ongun, Sibel Oguzlar

**Affiliations:** † Chemistry and Chemical Processing Technologies Department, Chemical Technology Program, Izmir Vocational High School, Dokuz Eylül University, Izmir 35210, Turkey; ‡ Center for Fabrication and Application of Electronic Materials, Dokuz Eylül University, Izmir 35210, Turkey

## Abstract

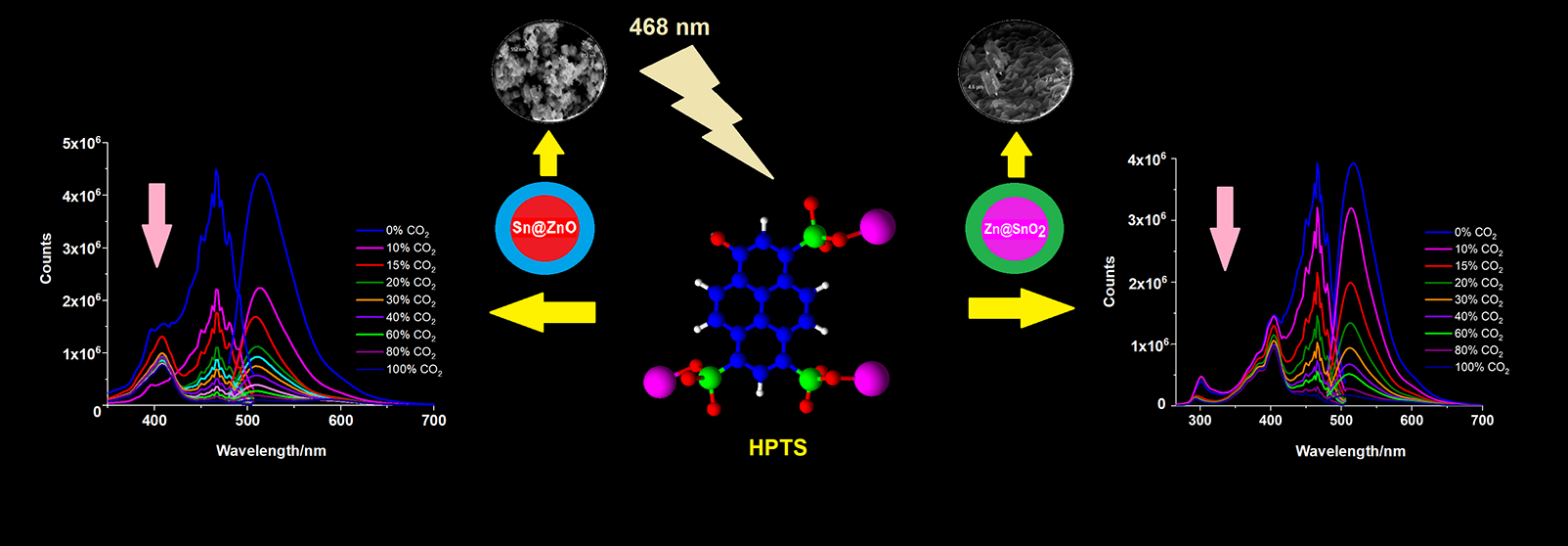

Fluorescent pH-sensitive indicator dye, 8-hydroxypyrene-1,3,6-trisulfonic acid (HPTS), has become known as a preferred alternative for continuous and accurate monitoring of dissolved and/or gaseous CO_2_ in chemistry, medical, and biochemical research. The objective of this work is to enhance the HPTS dye’s CO_2_ sensitivity in the presence of Zn@SnO_2_ and Sn@ZnO additive particles. Sol–gel synthesized metal oxide semiconductors (MOSs) were characterized using XRD, XPS, and SEM. The fluorophore dye and the MOS additives were embedded in the ethyl cellulose (EC) polymeric matrix to prepare the sensing thin films. The steady-state and decay kinetic measurements of the HPTS-based composites were obtained by PL spectroscopy for the concentration ranges of 0–100% p[CO_2_]. As expected, the addition of MOSs improves the sensor characteristics, specifically its CO_2_ sensing ability, linear response range, and relative signal change compared to the free form of HPTS. The CO_2_ sensitivities of the HPTS-based thin films were found at 17.6, 23.2, and 40.9 for the undoped, Zn@SnO_2,_-doped, and Sn@ZnO-doped forms of the HPTS, respectively. Additionally, the response and recovery times of the HPTS-based sensor agent with Sn@ZnO were measured as 10 and 460 s, respectively. The obtained results demonstrate that materials composed of HPTS with MOSs are potential candidates for CO_2_ sensors.

## 1 Introduction

In the fields of chemistry, medicine, and biochemistry, accurate measurement and monitoring of carbon dioxide (CO_2_) levels are important for understanding biological processes, studying enzymatic reactions, assessing metabolic activity, and evaluating the effectiveness of drugs or therapeutic interventions. Due to this, it is crucial that CO_2_ gas can be precisely and constantly measured over a broad area. Techniques based on infrared absorptiometry, electrochemistry, and luminescence can be used to primarily identify the presence of CO_2_ gas. Optical sensors have gained popularity in recent years as a result of their advantages, such as electrical isolation, minimal noise interference, lower cost, fast response, adaptability for miniaturization, and simplicity of production and use.^1−6^ Currently, electrochemical and optical devices based on spectroscopic changes are used to measure CO_2_, but optical chemical measurement methods have advantages, such as shorter response times, greater sensitivity, stability, and lower prices. The most common pH-sensitive fluorescent indicator dye, 8-hydroxypyrene-1,3,6-trisulfonic acid (HPTS), is commonly used for the accurate and continuous monitoring of dissolved CO_2_ gas in various research fields. It exhibits strong emission and excitation bands when excited at 468 nm. It is simpler to immobilize HPTS on appropriate polymeric support materials when it is used as a trisodium salt or an ion pair.^7−11^ In photoluminescence-based CO_2_ sensors, the studied pH-sensitive dye is physically trapped in polymeric matrices such as silicone, ethyl cellulose (EC), polystyrene (PS), and poly(methyl methacrylate) (PMMA), which provide a stable and protective environment for the dye molecules while allowing for interaction with the surrounding environment. Despite the widespread use of HPTS dye in optical applications and its superior response to CO_2_, it exhibits some disadvantages, such as stability, low relative signal variation, and reproducibility. As such, HPTS dye has been used with various additives such as ionic liquids (ILs),^12^ metal oxide nanoparticles (NPs),^1,2^ coordination polymers (CPs),^13^ and/or metal oxide semiconductors (MOSs)^14,15^ to eliminate such disadvantages. By incorporating MOSs into the sensor design, it is possible to increase the sensitivity and selectivity toward gas molecules. The porous structures of MOSs allow for a larger surface area available for gas adsorption, leading to enhanced sensing capabilities.^14,15^ Metal oxide semiconductors (such as ZnO, CuO, SnO_2_, TiO_2_, Co_3_O_4_, Fe_2_O_3_, and WO_3_), which have n-type and p-type forms, perform numerous gas sensing applications due to their chemical and physical properties and distinctive structure.^1,16−21^ MOSs-based gas sensors detect many target gases, including both oxidizing and reducing gases such as O_2_, CO_2_, CO, NO_2_, H_2_, H_2_S, SO_2_, and CH_4_ and solvent vapors such as ammonia, acetone, toluene, and ethanol.^22−25^ Various metal oxide doping materials such as a MXene/CuO composite driven by TENG, a TENG-driven MXene/TiO_2_/SnSe composite, a Pd-doped CoTiO_3_/TiO_2_ (Pd-CTT) nanocomposite, and a ZnO/SnSe_2_ composite film for ammonia, SO_2_, benzene, and CO gas sensor applications were reported by Wang et al.^26−29^

In core@shell systems, heteroatom doping with different metals can successfully increase the gas sensitivity of the MOSs in addition to altering the material with noble metals. The inclusion of these metal heteroatoms modifies the size, porosity, and specific surface area of MOSs, which, in turn, influences the adsorption sites of gas molecules and diffusion pathways. The presence of these heteroatoms can lead to changes in the crystal structure, resulting in a modified lattice size or unit cell dimensions. Changes in size, porosity, and surface area can affect the accessibility and connectivity of these diffusion pathways, altering the diffusion rates and pathways of gas molecules within the material.^22^ For applications involving gas sensing, non-noble metals have also been reported to be used as cores in metal core@metal oxide shells.^30^ In metal core@metal oxide shell nanoparticles, the basic metal atoms of an MOS are replaced by heteroatoms, resulting in a smaller grain size. By increasing the gas sensing ability of MOSs, gas sensors can be produced that outperform those composed of metal oxides.^22^ In recent years, metal core@metal oxide shell structures with SnO_2_ or ZnO as the shell for gas sensing applications have received much research. Rai and co-workers presented the Au@SnO_2_ core@shell structure for CO gas sensing applications and showed a greater response compared to pristine SnO_2_.^31^ In their study on the Au@SnO_2_ core@shell structure, Yu et al. claimed target gases like CO catalytically react with oxygen adsorbents. According to the electrical mechanism, the modulation of the Schottky barrier caused by the creation of depletion zones around the particles improves the sensing of CO.^31^ Wu et al. reported that SnO_2_, Ag/SnO_2_, and Ag@SnO_2_ structures were used to detect ethanol vapor. When exposed to 200 ppm ethanol gas, the response time (*t*_90_) and recovery time (*t*_R90_) of the SnO_2_ material were measured as 54 and 85 s, while those of the Ag@SnO_2_ material were 34 and 68 s, respectively.^32^ Chung et al. explained the production of HCHO gas-sensitive Au@SnO_2_ core@shell structures by the sol–gel method. While the SnO_2_ material did not react against HCHO gas, the 1 wt % Au-doped SnO_2_ material increased the sensor response by 2.4, while the Au@SnO_2_ material increased it by 2.9. It has also been reported that Au@SnO_2_ material reduced the response times.^33^ Li et al. created Au@ZnO NPs and compared their CH_2_O-sensing abilities to those of ZnO and 1 wt % Au-doped ZnO NPs. With a faster reaction and recovery time, Au@ZnO exhibited an improved response signal.^34^ Yang and co-workers reported that hydrothermal synthesized CdS/ZnO core@shell nanowires (NWs) demonstrated excellent visible-light-activated gas sensing performance toward ppb-level NO_2_ at room temperature, with responses ranging from 6.7% to 337% when exposed to NO_2_ concentrations from 5 to 1000 ppb.^35^ α-Fe_2_O_3_@ZnO core@shell NWs on the microelectromechanical system structure were prepared by Yang et al. for H_2_S gas detection. They have a maximum response to H_2_S of ∼1.1, while the pure α-Fe_2_O_3_ NW gas sensor response of 5.98 is approximately five times higher. Additionally, gas sensors based on α-Fe_2_O_3_@ZnO core@shell NWs exhibited selectivity and response/recovery features toward H_2_S.^36^ According to the literature, many gas sensor studies containing metal oxides are based on electrical measurement. The advantage of the luminescence-based measurement is that it is used as an alternative in the absence of electrical contact between the resulting impurities and nanostructures. In addition, real-time information about the variation of certain additives in the photoluminescence spectra can be observed. However, the effect of adsorbed gases on the photoluminescence of metal oxide powders has not yet been adequately studied. This situation motivated us to study photoluminescent gas sensing materials based on Zn@SnO_2_ and Sn@ZnO additives for the determination of CO_2_ gas levels. In the current study, the CO_2_ sensitivity of HPTS when used along with Zn@SnO_2_ and Sn@ZnO additives exhibited a considerable increase compared with the additive-free form. There has been an enhancement in the sensitivity and linear CO_2_ response of HPTS when used with Zn@SnO_2_ and Sn@ZnO additives. This improvement should be attributed to the gas adsorption on the surface of HPTS structures. However, when HPTS is used in the presence of Sn@ZnO NPs, the observed hypersensitivity to CO_2_ should be attributed to the increased surface defects and the high surface-to-volume ratio of the nanoparticles.

## 2 Materials and Methods

### 2.1 Materials

Zinc acetate dihydrate (Zn(CH_3_COO)_2_·2H_2_O; 99%), tin chloride dihydrate (SnCl_2_·2H_2_O; 98%), and potassium hydroxide (KOH; 85%) used in the synthesis of Sn@ZnO were of analytical grade and used without any additional purification. Chemicals used in the production of Zn@SnO_2_ consist of an ammonia solution (NH_3_), tin(IV) chloride pentahydrate (SnCl_4_·5H_2_O), hydrochloric acid (HCl), and zinc chloride hexahydrate (ZnCl_2_·6H_2_O; 99%). Tetrahydrofuran (THF), tetrabutylammonium hydroxide (TBAOH), dioctylphatalate (DOP) as a plasticizer, the ionic liquid (IL) 1-butyl,3-methyl imidazolium tetrafluoroborate [BMIM][BF_4_] that is used to increase stability, and ethyl cellulose (EC) that is used as a polymeric membrane were obtained from Sigma-Aldrich. N_2_ and CO_2_ gas cylinders were 99.9% pure and were obtained from Tinsa Gas, Izmir, Turkey. Tetraoctylammonium bromide (TOABr), dichloromethane (CH_2_Cl_2_), and 8-hydroxypyrene-1,3,6-trisulfonic acid trisodium salt (HPTS) were purchased from Sigma-Aldrich and were employed as ion pairs that had been prepared in earlier studies. In a solution of Na_2_CO_3_ (1 wt %) and CH_2_Cl_2_, the trisodium salts of HPTS and TOABr were combined in a 1:4 ratio. The production of the ion pair required the use of a separatory funnel, and the organic solvent was evaporated to obtain the ion pair.^12^

### 2.2 Instrumentation

The structural analyses of the produced nanopowder samples were performed with a Panalytical/Empyrean X-ray diffractometer (XRD) using CuKα radiation at a scanning rate of 0.01°/min. a Thermo Scientific Kα X-ray photoelectron spectroscope (XPS) with a beam size of 400 nm diameter and a monochromatic Al Kα X-ray source was used to analyze the surface chemistry and elemental content of the materials. Microstructure images were taken at different magnifications with a Zeiss Sigma 300 VP scanning electron microscope (SEM) to perform the morphological characterization. The steady-state photoluminescence (PL) measurements were performed using an FLSP920 Fluorescent Spectrometer. The measurement of the decay time values was carried out utilizing the time-related single photon counting mode (TCSPC) of the FLSP920. For detection CO_2_ measurements, CO_2_ and N_2_ gases were mixed with the Sonimix 7000A gas mixing system in a concentration range of 0–100%.

### 2.3 Synthesis of Zn@SnO_2_ and Sn@ZnO Core@Shell Structures

To synthesize Zn@SnO_2_ powder, 4 g of SnCl_4_·5H_2_O was dissolved in 200 mL of distilled water (DW) under vigorous stirring for 20 min. Aqueous NH_3_ was added dropwise to the solution to adjust the pH to 12. To the pH-balanced solution was added 1 g of ZnCl_2_·6H_2_O, and the mixture was stirred for 30 min. The precipitate was collected and washed with DW. After drying in the oven at 100 °C for 24 h, Zn@SnO_2_ crystals were obtained. In order to improve crystallinity, crystals were annealed for 2 h at 500 °C in an ambient atmosphere.^37^

For the preparation of Sn@ZnO powder, 0.5 M Zn(CH_3_COO)_2_·2H_2_O was dissolved in 50 mL of DW. SnCl_2_·2H_2_O prepared in a concentration of 0.1 M with 20 mL of water was added dropwise to the solution, and the reaction mixture was mixed for 30 min. Then, 2 M KOH in 50 mL of DW was added to the final mixture, and the mixture was stirred magnetically at room temperature. The resulting dispersions were purified several times with DW and ethanol to remove impurities. The final product was dried at 100 °C for 6 h and annealed at 300 °C for 3 h.^38^

### 2.4 Thin Film Preparation

The polymer-based sensing thin films were prepared by mixing 100 mg of EC as a polymeric matrix, 96 mg of DOP as a plasticizer, 24 mg of [BMIM][BF_4_] as an ionic liquid, 2.5 mL of THF as a solvent, and 0.10 mg of HPTS dye as a fluorophore dye. To enhance the CO_2_ sensitivity of the HPTS dye, 0.10 mg of Sn@ZnO and 0.10 mg of Zn@SnO_2_ powders were added separately to the HPTS-based polymeric cocktails. The compositions of HPTS-based cocktails in the absence and presence of Zn@SnO_2_ and Sn@ZnO additives are shown in Table 1. Herein, the prepared composites were spread on a polyester (Mylar TM type) support using the knife coating method, and the thickness measurements of thin films were performed with a Tencor Alpha Step 500 Profilometer. The average of the thickness measurement results was found to be 6.53 ± 0.04 mm (*n* = 5). Optical CO_2_ measurements were performed after the prepared HTPS-based thin-film sensor agents were placed in a quartz cuvette with a septum. A gas mixing system capable of producing precise CO_2_ concentrations was used. The different CO_2_ concentrations were given to the system by immersing a diffuser needle in the sensing agents under working ambient conditions. Before the HTPS-based thin films were exposed to CO_2_ gas, the gas was passed through thermostated wash bottles containing water heated to 25 °C and maintained at a constant relative humidity level of 100% to humidify it.

**Table 1:** Cocktail Compositions of the CO_2_-sensitive HPTS-Based Composites

					additive	
dye	EC (mg)	DOP (mg)	THF (mL)	IL (mg)	Sn@ZnO(mg)	Zn@SnO_2_(mg)
HPTS (0.10 mg)	100	96	2.5	24		
	100	96	2.5	24	0.10	
	100	96	2.5	24		0.10

## 3 Results and Discussion

### 3.1 Characterization of Sn@ZnO and Zn@SnO_2_

**Figure 1:**
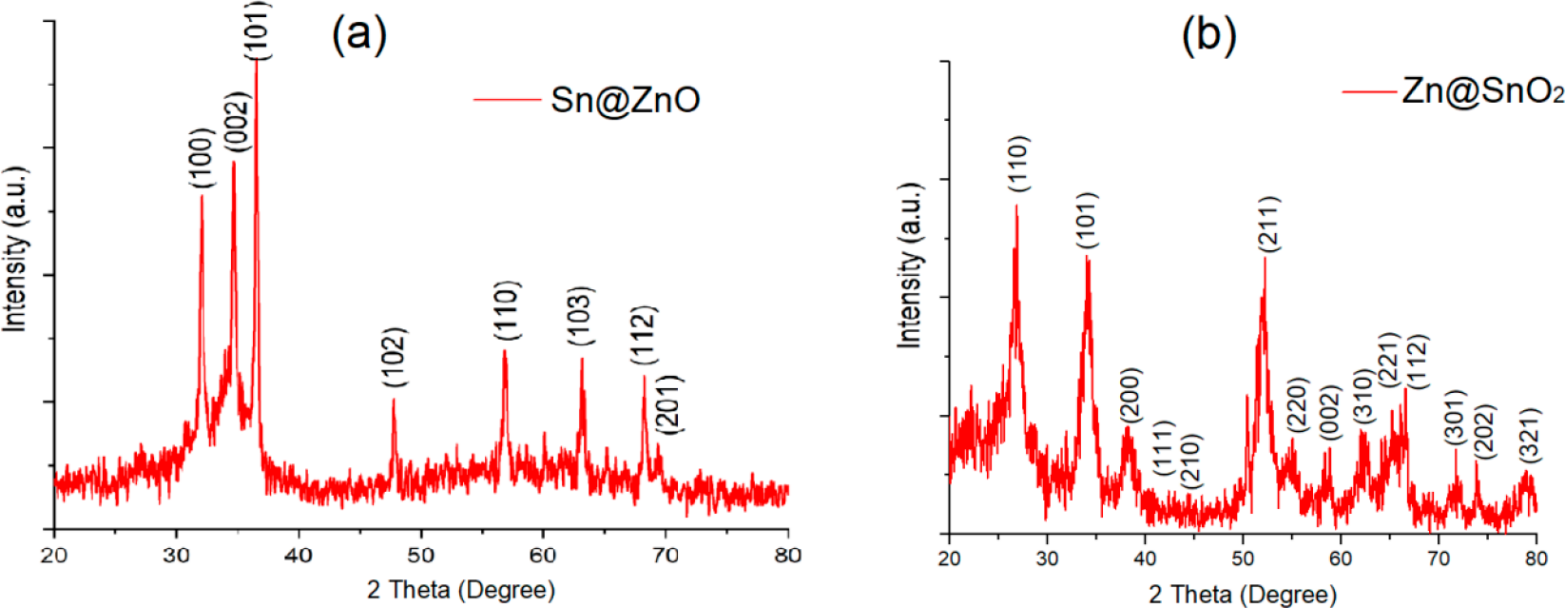
XRD diffraction spectra of (a) Sn@ZnO and (b) Zn@SnO_2_ crystals.

Figures 1 displays the results of an XRD analysis of both Sn@ZnO and Zn@SnO_2_ powders synthesized using the sol–gel method. ZnO crystals were shown to correspond to the JCPDS 36–1451 zincate model in (100), (002), (101), (102), (110), (103), (112), and (201), and the samples had a polycrystalline wurtzite structure. There was no sign of any impurity’s distinctive peak.^39^ The tetragonal phase planes of SnO_2_ were represented by characteristic peaks that can be found at different diffraction angles (110), (101), (200), (111), (210), (211), (220), (002), (310), (221), (112), (301), (202), and (321). All peak positions matched the normal SnO_2_ data very well (ICDD Card no. 01-0657). Additionally, neither impurity peaks with elemental zinc nor those for other zinc compounds were seen, demonstrating the effective integration of zinc atoms into the SnO_2_ crystal lattice.^40^

**Figure 2:**
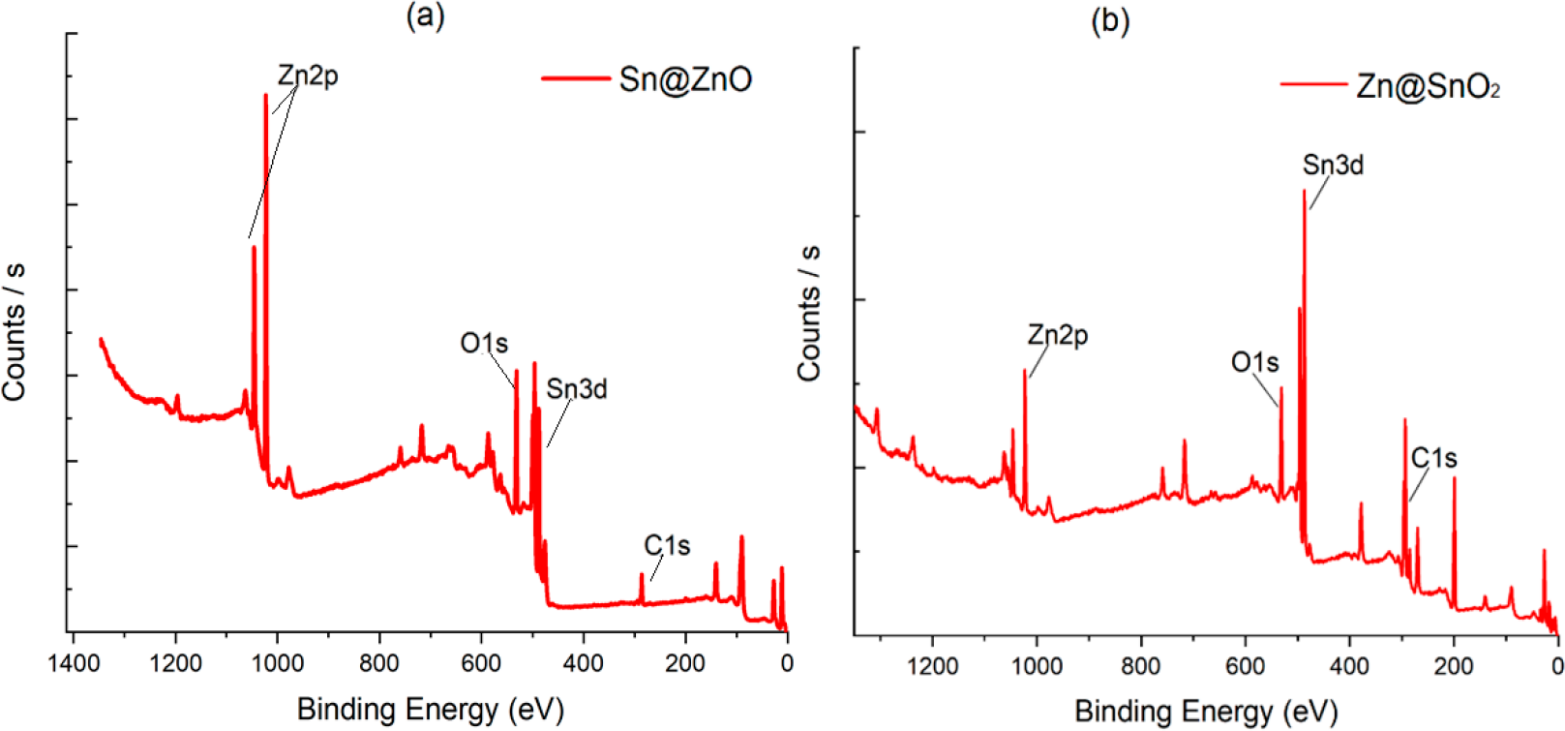
XPS scanning spectra of (a) Sn@ZnO and (b) Zn@SnO_2_ crystals.

The XPS spectra are given in Figure 2a and b, and Table 2 shows the results of both synthesized powders. Binding energies were calibrated using the C 1s hydrocarbon peak at ∼284 eV. Zn 2p was completely consistent with the divalent oxidation state of Zn^2+^, since the 2p^3/2^ and 2p^1/2^ peaks were separated at ∼1022 and ∼1043 eV, respectively. Additionally, the O 1s spectrum results highlight the two distinct oxygen atoms that were discovered at ∼530 and ∼531 eV. The binding energy of Sn 3d can vary depending on the specific oxidation state and chemical environment of the tin species being analyzed. The binding energy of Sn 3d is typically around 486–489 eV. The results of the Sn 3d spectrum at ∼486 and ∼494 eV were shown to correspond to Sn 3d^5/2^ and Sn 3d^3/2^, respectively (see Table 2).^41,42^

**Table 2:** Binding Energy (BE) and Atomic Weight (%) Values of Sn-Doped ZnO Crystals^a^

sample	element	binding energy (eV)	atomic weight (%)
Sn@ZnO	C 1s	285.79	9.25
	Sn 3d	487.31	20.47
	Zn 2p	1022.41	49.99
	O 1s	531.26	20.29
Zn@SnO_2_	C 1s	284.93	9.35
	Sn 3d	486.81	42.18
	Zn 2p	1022.31	24.85
	O 1s	530.96	23.62

a According to the XPS scanning spectrum.

**Figure 3:**
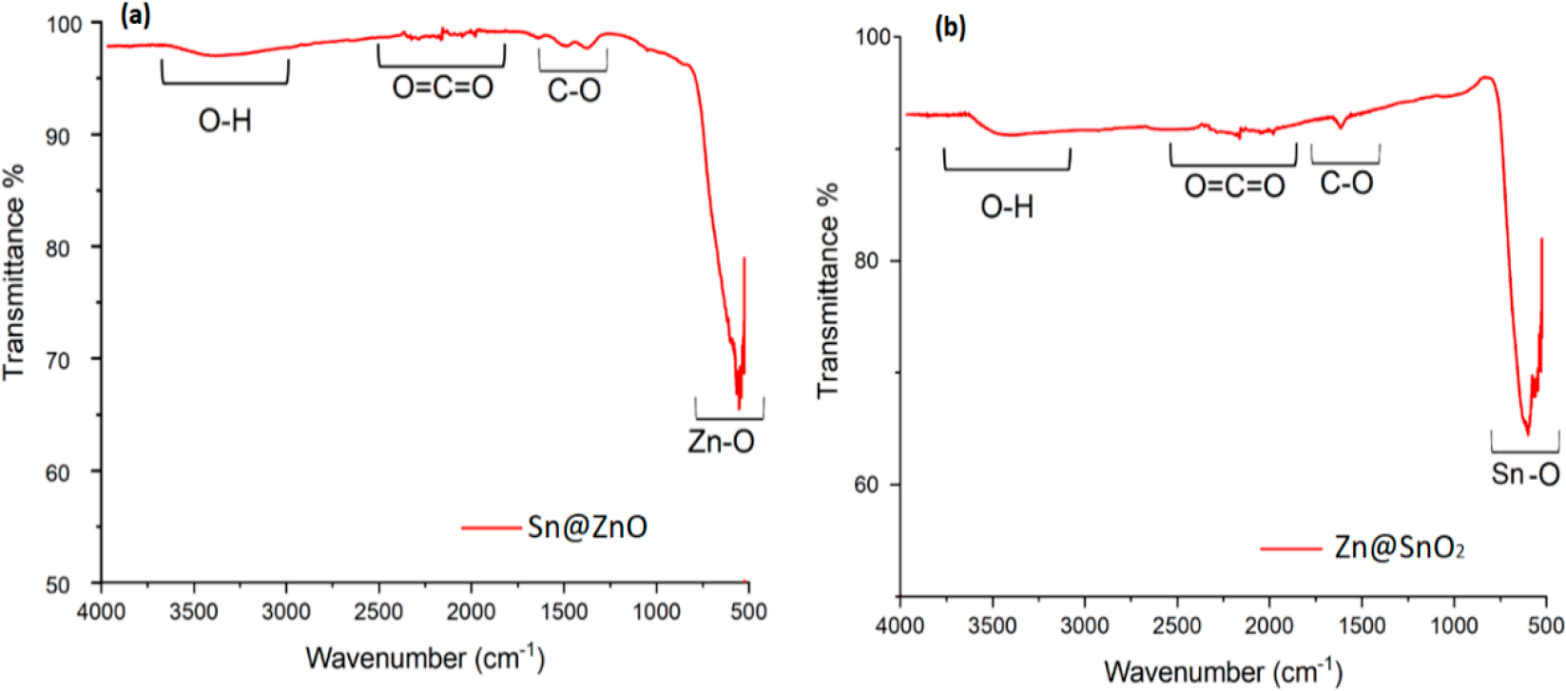
FTIR spectrum of (a) Sn@ZnO and (b) Zn@SnO_2_ crystals.

Figure 3 shows the FTIR spectra of the synthesized MOS powders. While the strong and broad band centered at 627 cm^–1^ corresponds to the Sn–O–Sn stretch, the peak of 628 cm^–1^ was due to the Zn–O–Zn vibration. In the literature, the characteristic stretching vibrations of ZnO bonds were observed in the absorption peak of wavenumbers between 500 and 650 cm^–1^.^43,44^ The broad band between 750 and 500 cm^–1^ is from the vibrations of Sn–O; −OH stretching vibrations in the wavenumber range of 3600 cm^–1^ are also due to the presence of H_2_O in the ZnO structure. It has been determined that there is a peak around 2340 cm^–1^ due to acetate and CO_2_ molecules in the air. There are symmetrical and asymmetrical stress modes belonging to the acetate group (−COOH) in the 1374 cm^–1^ wavenumber range. Metal oxide semiconductors usually have an absorption band below 1000 cm^–1^ due to interatomic vibrations.^37^

**Figure 4:**
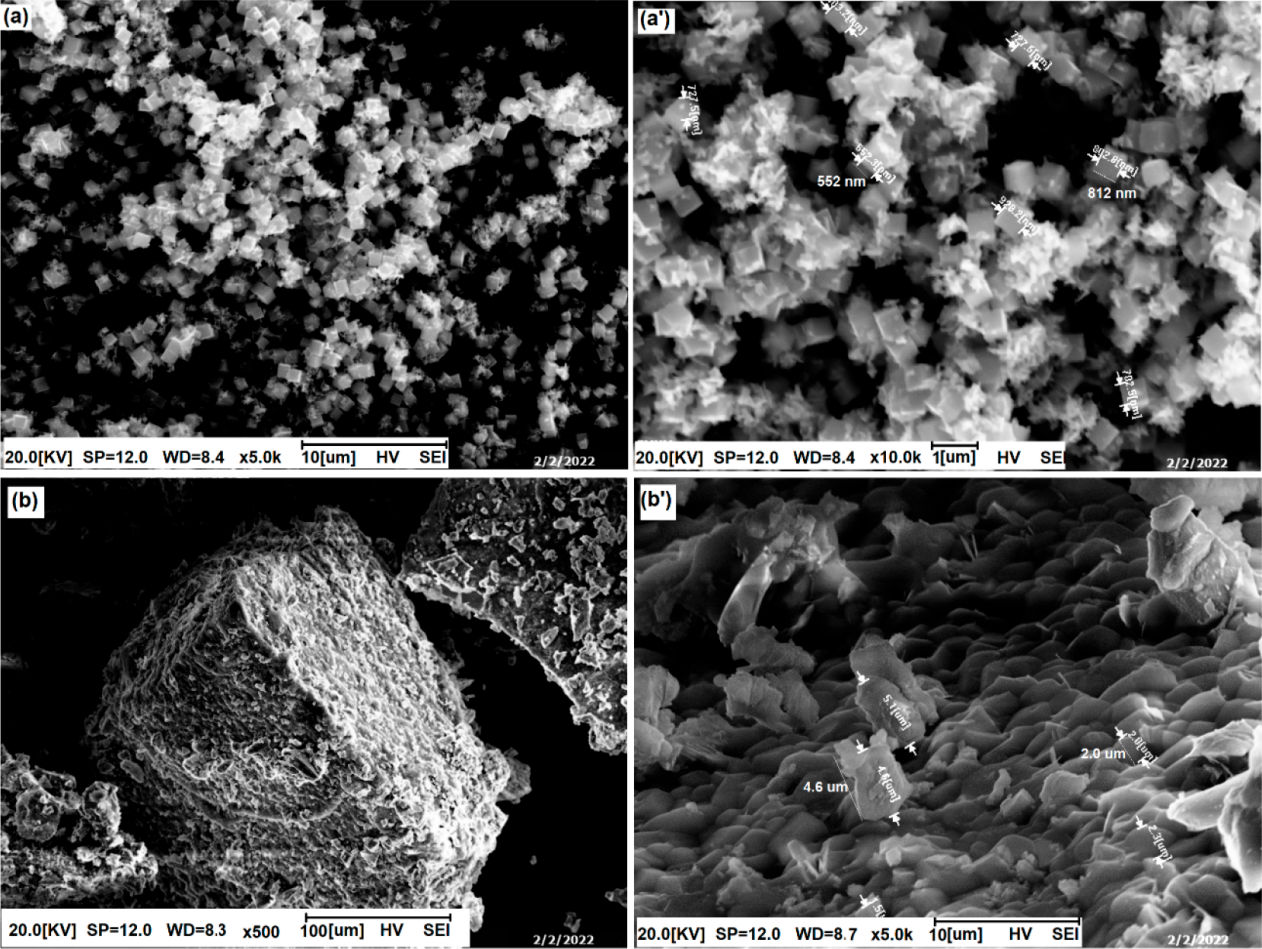
SEM images of (a and a′) Sn@ZnO and (b and b′) Zn@SnO_2_ crystals under different magnifications.

The surface morphologies of Sn@ZnO and Zn@SnO_2_ particles were characterized by SEM at different magnitudes. The hexagonal plate-like structures of Sn@ZnO nanoparticles range in size from 552 to 812 nm. While the structure consisted of Zn@SnO_2_ microparticles, some agglomerations were observed on the surface. The homogeneous distribution of these agglomerations provided good surface morphology (Figure 4).

### 3.2 CO_2_ Sensitivity of HPTS-Based Sensing Agents along with Sn@ZnO and Zn@SnO_2_

In this study, the increase in the CO_2_ sensitivity of HPTS with the use of Zn@SnO_2_ and Sn@ZnO powders as additives in the EC matrix was investigated. Measurements based on the signal change in the emission spectra of the prepared thin films were obtained after exposure to concentrations of 0–100% p[CO_2_] with the humidification of the gas. The humidification process of the CO_2_ gas is necessary for the formation of carbonic acid that reaches the active regions of the HPTS. With the increase in the CO_2_ concentration in the atmosphere, the dye changes into a less fluorescent form from green to light yellow and signal drops in the emission band at 515 nm are observed. The Stern–Volmer equation is widely used to describe the assumption of a dynamic quenching process where the quenching species interacts with the excited state of the fluorophore, reducing its emission intensity (eq 1).
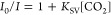
1where *I*_0_ is the intensity of the fluorescence or phosphorescence in the absence of the quencher, *I* is the intensity of the fluorescence or phosphorescence in the presence of the quencher at concentration [CO_2_], and K_SV_ is the Stern–Volmer constant.^10^

**Figure 5:**
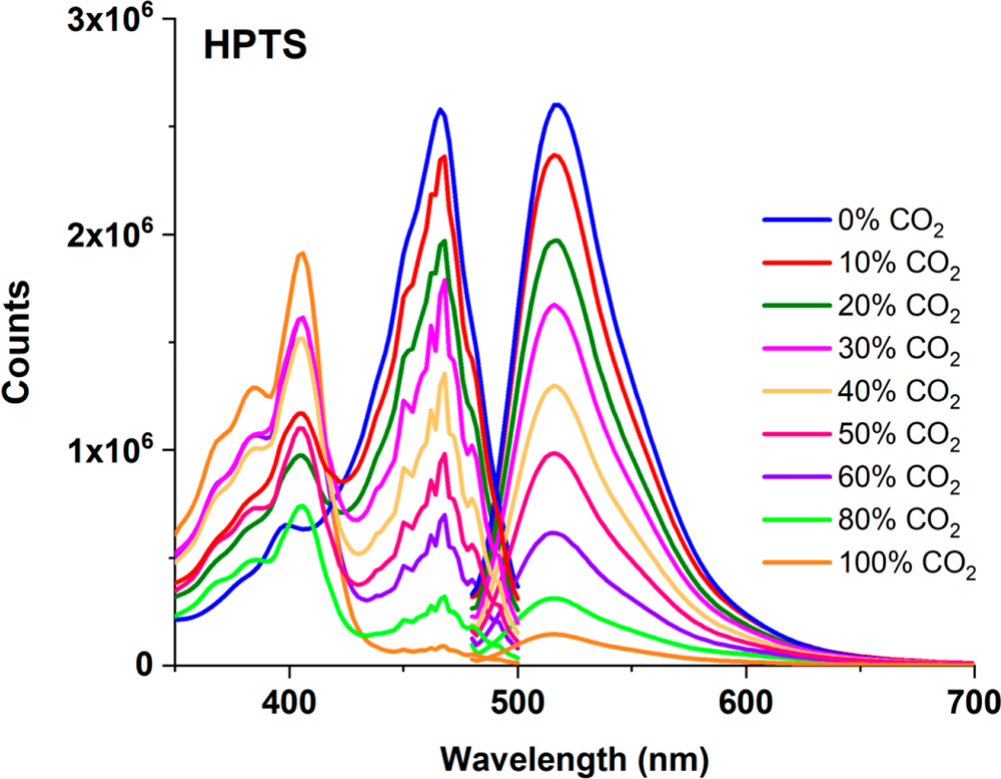
Emission-excitation spectrum of the free HPTS-based composite in an EC thin film for the 0–100% p[CO_2_] concentration range.

The CO_2_-induced spectrum behavior of additive-free HPTS in the 0–100% p[CO_2_] concentration range is shown in Figure 5. Based on the excitation of thin films in the EC matrix at 467 nm, signal decreases were observed in emission intensities at 515 nm.

**Figure 6:**
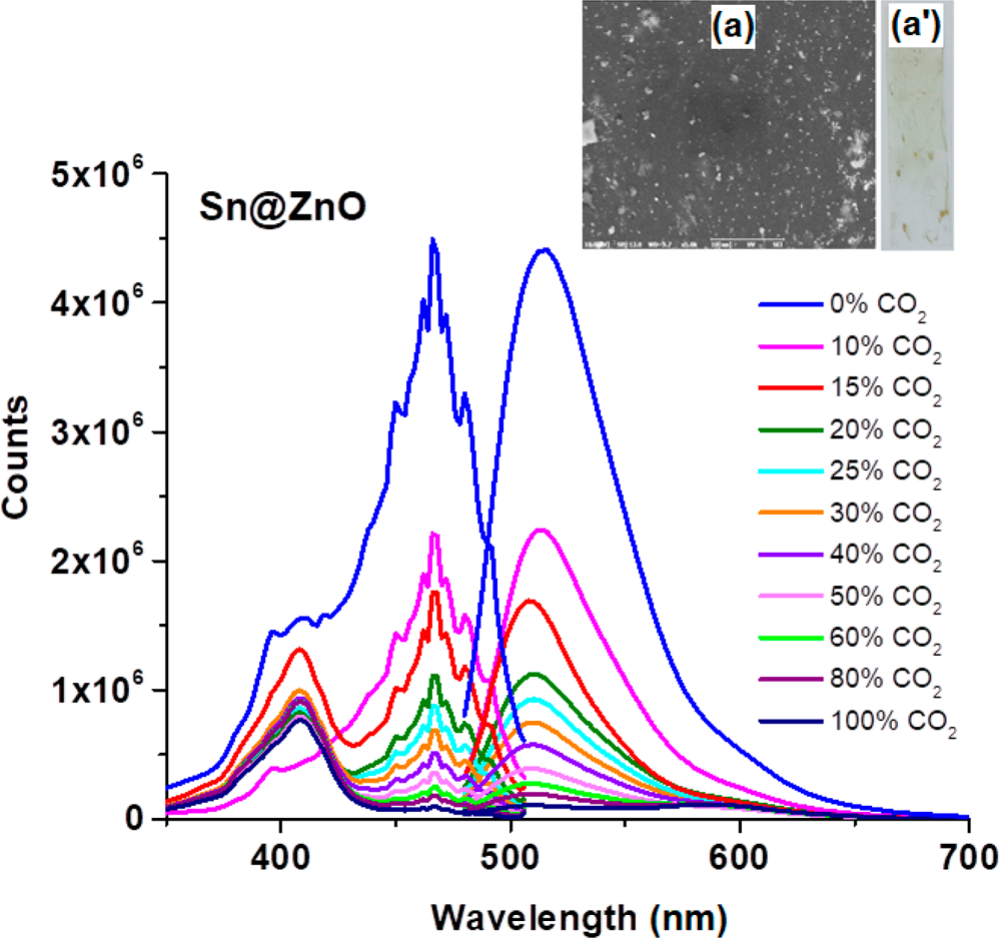
Emission-excitation spectrum of the Sn@ZnO additive-doped HPTS-based composite in an EC thin film for the 0–100% p[CO_2_] concentration range. Insets: SEM images of (a) a thin film and (a′) the thin film of the Sn@ZnO additive-doped HPTS-based composite.

**Figure 7:**
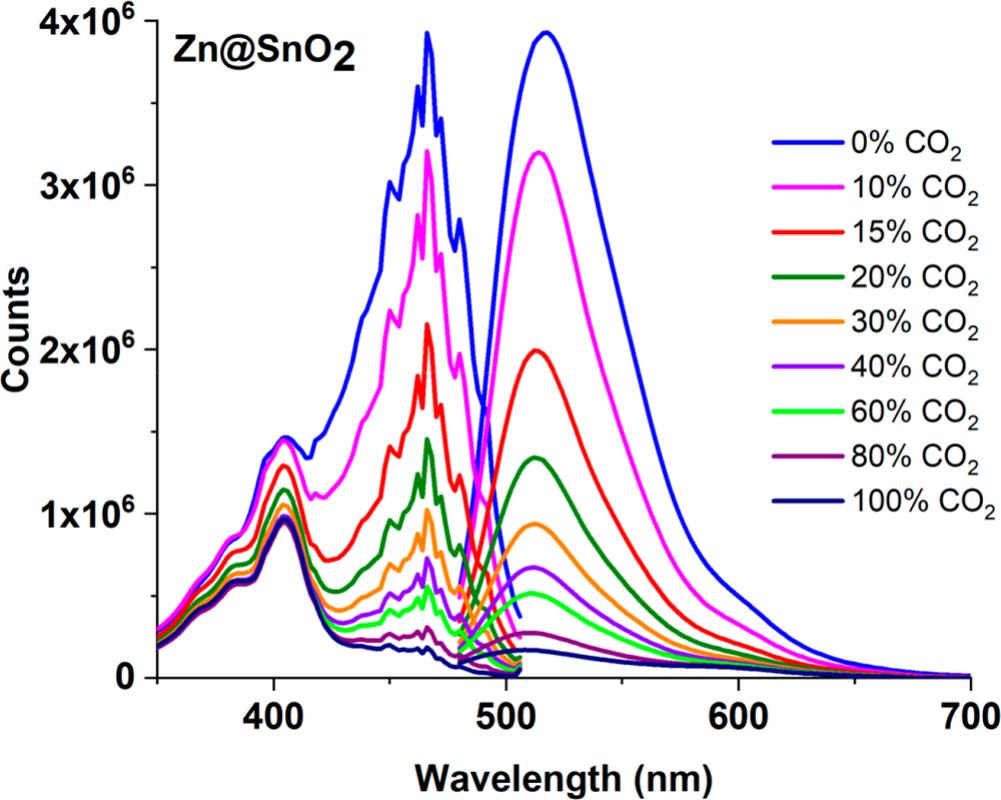
Emission-excitation spectrum of the Zn@SnO_2_ additive-doped HPTS-based composite in an EC thin film for the 0–100% p[CO_2_] concentration range.

Figures 6 and 7 reveal the CO_2_-induced variations of the absorption and emission spectra of the HPTS-based composite in the presence of Sn@ZnO and Zn@SnO_2_ additives, respectively.

**Figure 8:**
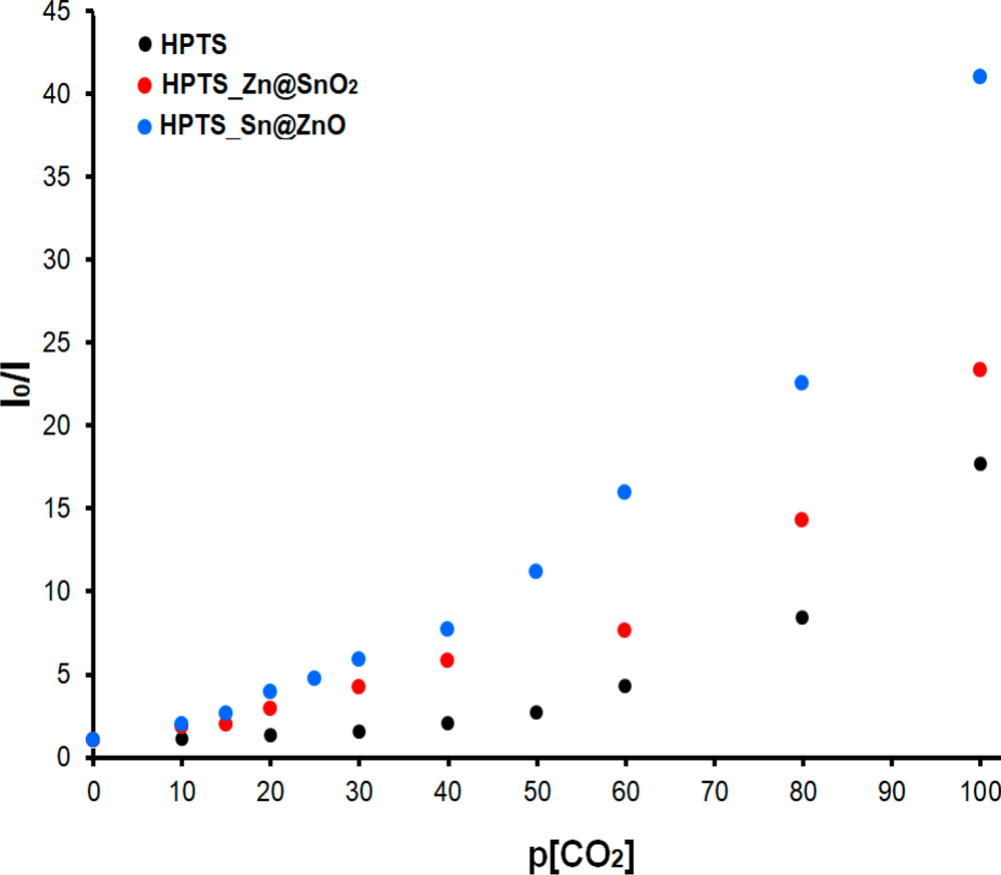
Comparative calibration curves of all HPTS-based composites for the 0–100% p[CO_2_] concentration range.

The calibration graphs of all analyzed thin films in the concentration range of 0–100% p[CO_2_] are given in Figure 8 comparatively. Table 3 shows the calibration equation, the Stern–Volmer (*K*_SV_) constant, the regression coefficient, and the sensor sensitivity (*I*_0_/*I*_100_) values of all HPTS-based composites obtained due to the calibration graphics. The correct equation for the indicated CO_2_ concentration range of the additive-free HPTS sensor slide was calculated as *y* = 0.0217*x* + 1, and the correlation coefficient was 0.9128. However, for the concentration range of 0–40% p[CO_2_], the correct equations of HPTS_Sn@ZnO and HPTS_Zn@SnO_2_ composites were found to be *y* = 0.1558*x* + 1 and *y* = 0.1089*x* + 1, and the correlation coefficients were 0.9815 and 0.9505, respectively. When the CO_2_-induced variations of the composites were examined, it was observed that the HPTS_Sn@ZnO sensor material had a considerably higher slope and superior linear response in the 0–100% p[CO_2_] range compared to other composites.

**Table 3:** Optical Properties and CO_2_ Sensitivities of Hpts-Based Composites

sample	equation (0–40% p[CO_2_])	Stern–Volmer constant (*K*_sv_)	*R*^2^	*I*_0_/*I*_100_
HPTS	*y* = 0.0217*x* + 1	2.17 × 10^–2^	0.9128	17.6
HPTS_Sn@ZnO	*y* = 0.1558*x* + 1	1.56 × 10^–1^	0.9815	40.9
HPTS_Zn@SnO_2_	*y* = 0.1089*x* + 1	1.09 × 10^–1^	0.9505	23.2

In this study, the use of Sn@ZnO and Zn@SnO_2_ crystals with HPTS in the EC matrix resulted in improved *I*_0_/*I*_100_ values compared with previous studies in the literature. It was seen that the enhancement of the CO_2_ sensitivity was a result of the addition of MOSs to the dye as an additive. While the *I*_0_/*I*_100_ value indicating the sensor sensitivity for HPTS without additives was 17.6, these values were found to be 23.2 and 40.9 with Zn@SnO_2_ and Sn@ZnO additives, respectively (Table 3). It was observed that HPTS_Sn@ZnO thin films increased the sensitivity to carbon dioxide gas ∼10× compared to the additive-free form.

In comparison to the free form of HPTS-based composites, the MOS-doped HPTS-based sensing slides have a quick response time and better sensitivity. In many instances, heteroatom doping with different metals can also successfully increase the gas sensitivity of MOSs. The inclusion of these metal heteroatoms modifies the size, porosity, and specific surface area of the MOS, which in turn alters the adsorption sites and diffusion pathways of gas molecules. It has been known that the surface shape of the MOS structures changes when metal additives are added as core@shell structures. As can be seen from the results obtained by SEM analysis, the Sn@ZnO additive showed higher sensitivity, since it has a smaller grain size than the Zn@SnO_2_ additive. Since nanoparticles have a large surface area and hence more chemisorbed oxygen ions and a higher barrier height, the gas sensing capability can be improved.^24,45−47^

### 3.3 Interactions between HPTS and MOSs

**Figure 9:**
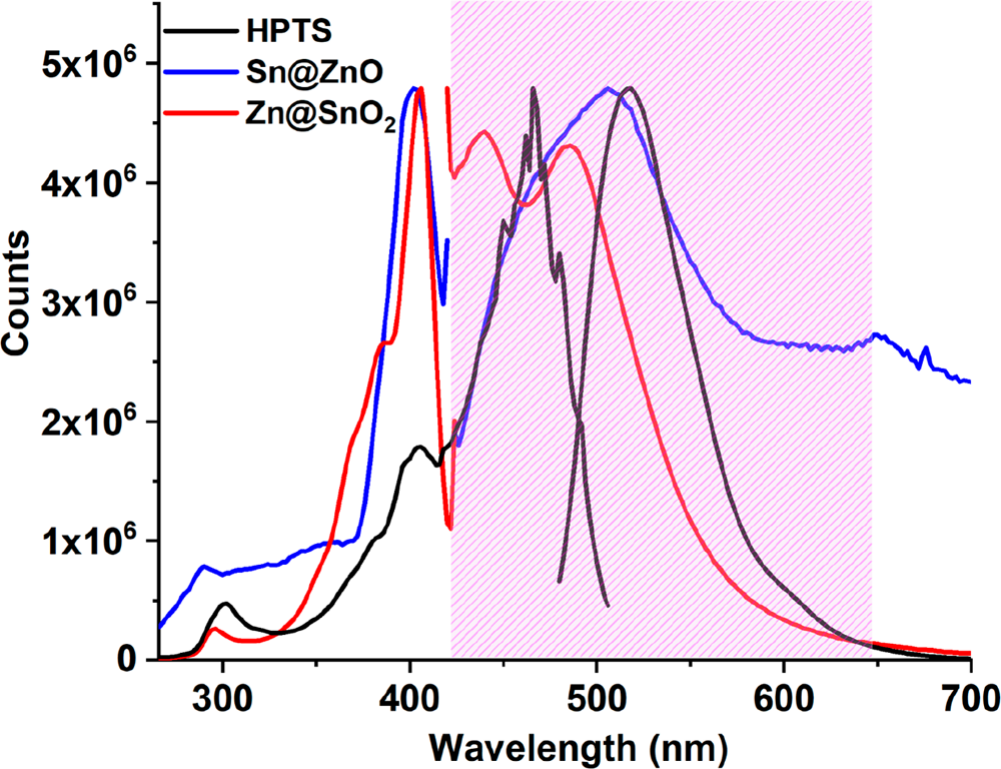
Excitation-emission spectra of EC-embedded HPTS, Sn@ZnO, and Zn@SnO_2_ excited at their own excitation wavelengths.

**Figure 10:**
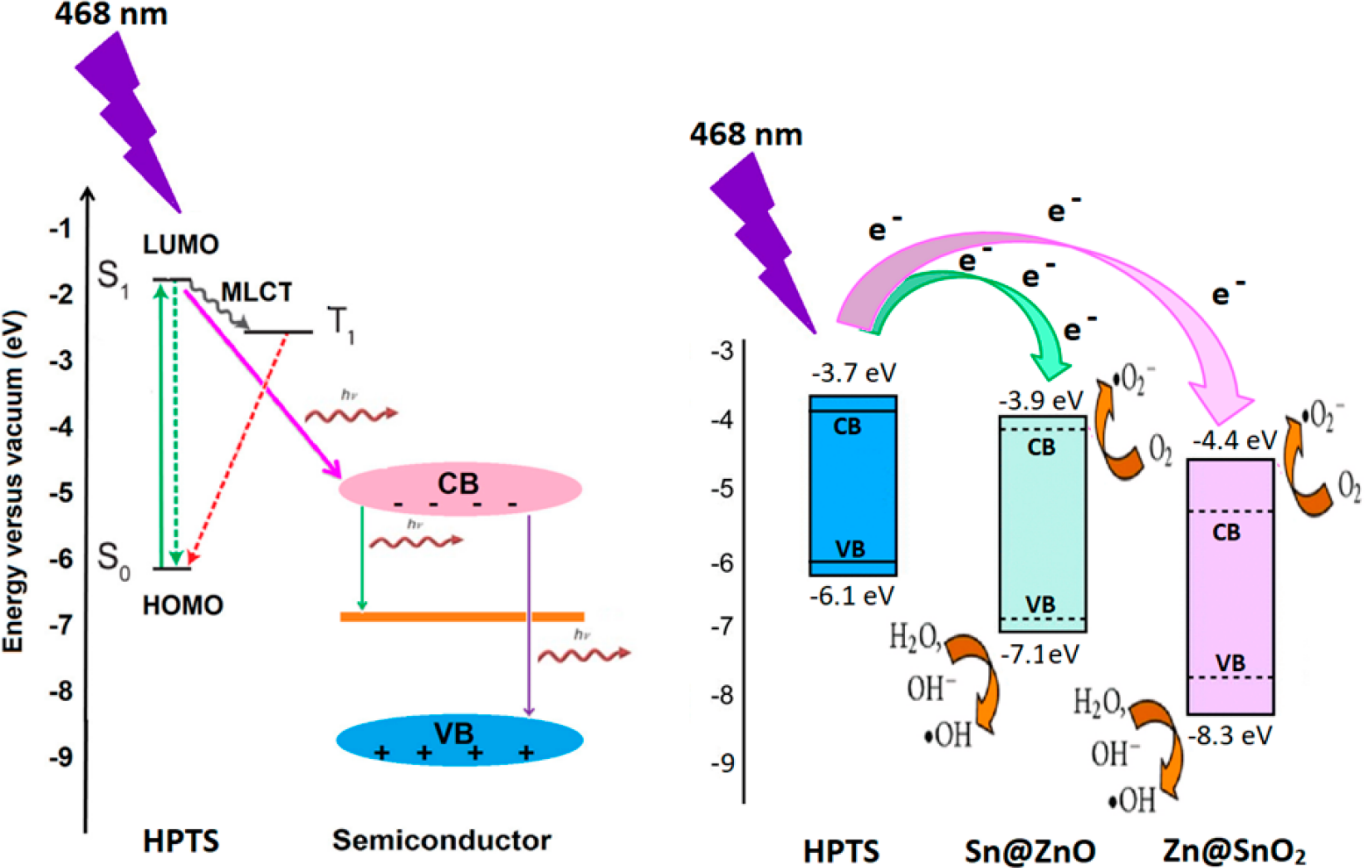
Energy level diagram of the electronic states of HPTS, Sn@ZnO, and Zn@SnO_2_.

To better understand the interaction causing the increase in the CO_2_ sensitivity, we separately recorded the excitation and emission spectra of the HPTS, Sn@ZnO, and Zn@SnO_2_ particles when excited at 370 and 468 nm (Figure 9). One of the probable reasons for the observed enhancement may be energy transfer between the MOSs and the HPTS, since both the Sn@ZnO and Zn@SnO_2_ additives are substantially absorbed between 270 and 430 nm and releas at a broad wavelength range between 420 and 700 nm, covering the HPTS excitation band. These ranges overlap with the excitation band of HPTS, suggesting the possibility of energy transfer between the metal oxide particles and HPTS. Energy transfer, also referred to as Förster resonance energy transfer (FRET), is the term used to describe the phenomena. FRET occurs when two fluorophores (light-absorbing molecules) are in close proximity, and the excitation energy from one fluorophore is transferred to the other through nonradiative dipole–dipole interactions. In this case, the MOSs and HPTS act as potential energy donors and acceptors, respectively. The higher-energy electron in the lowest unoccupied molecular orbital (LUMO) of the excited fluorophore can undergo a transition to the conduction band (CB) of metal oxide semiconductors, leading to electron transfer between the two systems. Regarding the increase in fluorescence quenching of HPTS in the presence of a CO_2_ atmosphere, the transfer of electrons from the excited singlet state to the CB of metal oxide semiconductors may contribute to this phenomenon. The presence of CO_2_ can lead to energy dissipation pathways, such as electron transfer to gas molecules or other reactive species, resulting in the quenching of fluorescence.^48^Figure 10 shows the schematic diagram of energy transfer between HPTS and MOSs. The enhanced CO_2_ sensitivity of the Sn@ZnO NPs may be attributed to the broad overlap of the emission band of the Sn@ZnO NPs with both the absorption and emission bands of HPTS. The mechanism underlying the strong absorption and emission abilities of ZnO involves band gap excitation of the crystal by energetic photons, which produce exciton pairs with holes in the valence band and electrons in the conduction band. When CO_2_ molecules adsorb onto the metal oxide surface, they can either donate or accept electrons, causing changes in the electrical conductivity of the material. It can then interact with metal oxide surfaces, collect electrons from the conduction band, and facilitate the production of oxygen ions (O_2_^–^, O^–^, or O_2_). In the absence of light irradiation, electrons are captured from the conduction band and produce oxygen species. Meanwhile, other oxygen ions, including O^–^ and O_2_, may develop on the surface as a result of light exposure. Light irradiation increases the number of charge carriers on the surface because photoelectrons are created, which improves oxygen adsorption and the interaction of oxygen ions with CO_2_ molecules, which improves the surface adsorption and desorption of CO_2_ gas to the sensor surface.^49,50^

Both HPTS and metal oxide particles competed with each other for absorbance when immobilized in the proximity of the polymeric matrix and simultaneously excited by the light source. For HPTS, metal oxide particles likely act as light-collecting centers, creating new opportunities for additional excitation. However, together with FRET energy transfer, emission from metal and oxygen vacancies, interstitial metal ions, oxygen anticides, and electronic transitions between interstitial metal ions during relaxation improves the emission performance of HPTS. This is due to the efficient transfer of electrons from the excited state of HPTS to the conduction band of the semiconductor nanoparticle (Figure 10).^51−61^

### 3.4 Decay Time Measurements

**Figure 11:**
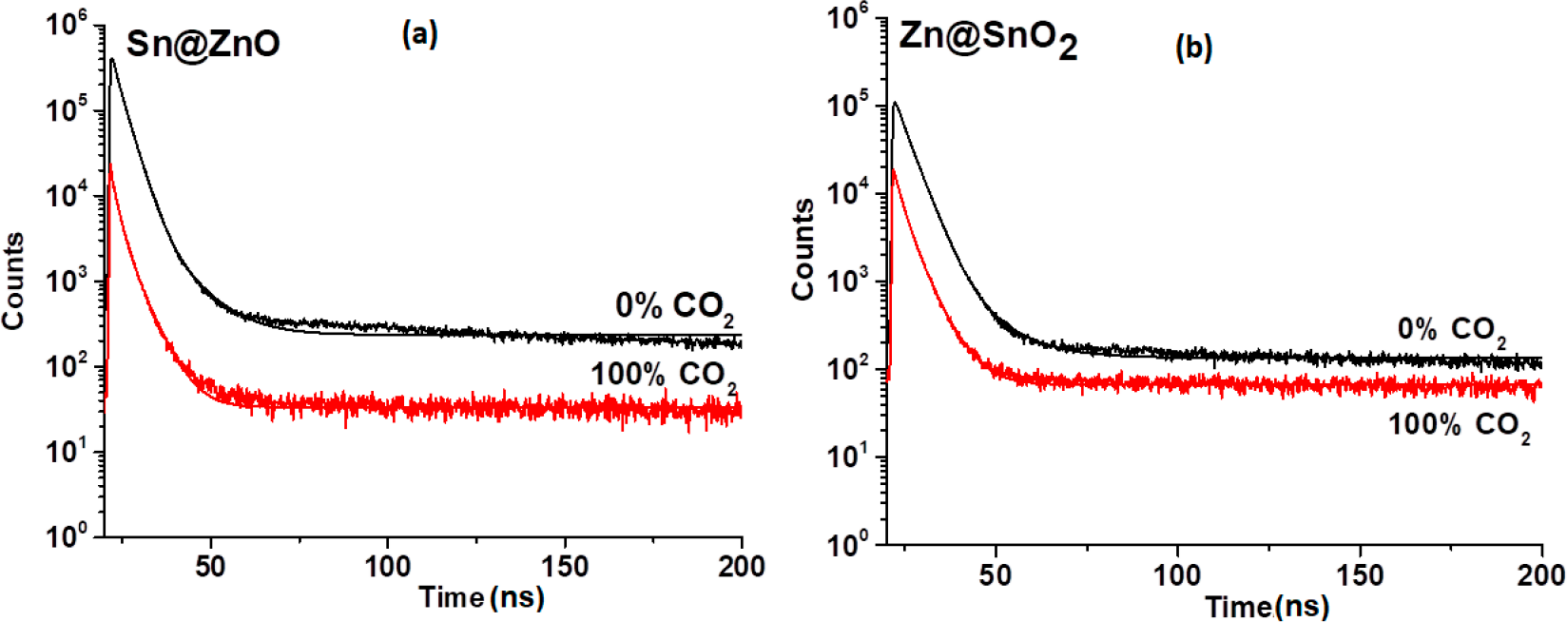
Decay curves of (a) HPTS_Sn@ZnO and (b) HPTS_Zn@SnO_2_ in the fully N_2_ and CO_2_ gas atmospheres.

Longer or shorter decay times in the presence of CO_2_ can indicate changes in the fluorescence dynamics such as energy transfer, quenching, or other interactions occurring due to the presence of CO_2_ molecules. The decay time values were measured for the HPTS dye when combined with the Sn@ZnO and Zn@SnO_2_ additives. Table 4 shows all of the recorded decay time values. Measuring the decay kinetics of HPTS-based thin films gives us important information about the interaction mechanism between the quencher and fluorophore (see Figure 11). While the fluorescence decay time values of HPTS_Sn@ZnO and HPTS_Zn@SnO_2_ were recorded as 4.26 and 3.46 ns in the N_2_ atmosphere, all of the phosphorescence decay time values were reduced to 3.71 and 3.12 ns, respectively, when fully exposed to CO_2_ (see Table 4). When combined with Sn@ZnO and Zn@SnO_2_ additives, the multiple exponential decay time values of HPTS exhibited a greater decrease than those of the additive-free forms. The obtained results showed that factors such as surface defects, electrical conductivity, and charge transfer cause a reduction in decay time kinetics. The adsorbed or diffused gas in the MOSs decreases the carrier density, resulting in potential barriers between oxide particles that cause reduced electrical conductivity. This charge mobility also causes a decrease in the luminescence intensity and decay time kinetics.

**Table 4:** Decay Time Values of the HPTS-Based Sensing Agents

sample	τ_O_ (0% CO_2_)	decay time (ns)	std. dev. (ns)	rel. (%)	τ_O_ (100% CO_2_)	decay time (ns)	std. dev. (ns)	rel. (%)
HPTS_Sn@ZnO	τ_1_	3.76	0.008	92.74	τ_1_	2.27	0.043	48.36
	τ_2_	10.66	0.203	7.26	τ_2_	5.06	0.077	51.64
	τ_avr_		**4.26**		τ_avr_		**3.71**	
HPTS_Zn@SnO_2_	τ_1_	2.89	0.003	90.22	τ_1_	1.64	0.023	49.12
	τ_2_	8.77	0.069	9.78	τ_2_	4.55	0.047	50.88
	τ_avr_		**3.46**		τ_avr_		**3.12**	

### 3.5 Reproducibility of the Sensor Slides

**Figure 12:**
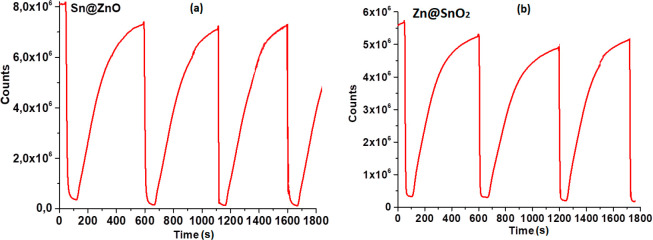
Kinetic response of both (a) HPTS_Sn@ZnO and (b) HPTS_Zn@SnO_2_ composites after exposure to certain CO_2_ concentrations.

The response and recovery time for gas sensing refers to the time it takes for a CO_2_ gas sensor to detect the presence of CO_2_ and then return to its baseline reading after the CO_2_ concentration has changed. The MOS additives assist in the desorption of CO_2_ molecules from the sensing material, improving the recovery time of the HPTS and its ability to repeat the sensing cycle. The regeneration measurement is a crucial parameter for CO_2_ sensor applications, as it determines the sensor’s ability to recover its sensing properties after exposure to CO_2_. The response and reversibility measurements of the EC thin film-embedded HPTS dye in the presence of MOSs were interpreted according to time and varying quencher concentrations in fully N_2_ and CO_2_ gas atmospheres. The results obtained were response time and reversibility for CO_2_ sensing (see Figure 12). The signal changes of HPTS-based thin films with Sn@ZnO and Zn@SnO_2_ were reversible during measurements after the third cycle, and the standard deviations of the upper and lower signal intensities were found to be less than 5.0%. The response and the recovery times of HPTS_Sn@ZnO and HPTS_Zn@SnO_2_ were determined as 10, 20, 460, and 520 s in fully CO_2_ and N_2_ atmospheres (see Figure 12).

The long-term stability of fluorescent dye is one of the important factors in optical sensor design. Since the long-term instability of HPTS-based optical sensors is an important factor to be improved, in the study, we used an ionic liquid during the preparation of thin films to improve the stability of HPTS. Even after 10 months, the HPTS-based sensing slides stored in the dark under laboratory conditions showed nearly the same emission-based intensity. Considering the results obtained in this study, we confirmed that all sensing membranes gave reproducible and stable responses for CO_2_ measurements.

## 4 Conclusion

As a result, herein, the pH-sensitive HPTS dye was used in the presence of additives of Sn@ZnO and Zn@SnO_2_ for the first time, and thus HPTS dye has been shown to increase its sensitivity to CO_2_ gas. Although the *I*_0_/*I*_100_ value with the additive Zn@SnO_2_ was 23.2, this value increased 40.9-fold in the case of the Sn@ZnO additive. The results show that doping metal oxides with non-noble metals generates free electrons that facilitate the adsorption of CO_2_ molecules on the surface and defect and morphology changes. Analysis based on measurement of the emission intensity with the addition of additives showed that HPTS showed a higher sensitivity to CO_2_ gas, a higher *K*_sv_ constant, and a better relative signal change. In addition, data were obtained with a better linear calibration graph compared to HPTS without additives in the range of 0–100% p[CO_2_]. The charge transfer in the adsorption process is the basis of the gas sensing mechanism. As a result of the metal-doped semiconductor metal oxide addition, the results showed that these particles are among the most promising materials for CO_2_ sensor design. The obtained results can be attributed to defects in the semiconductor metal oxide system. Surface defects play a crucial role in the adsorption of gas molecules on oxide surfaces, inducing charge transfer between adsorbents and substrates even in the presence of a small number of defects. This shows that the material has important effects on its electronic, structural, and optical properties.

## Data Availability

Data will be made available on request.
